# A Novel Classification Algorithm Based on Incremental Semi-Supervised Support Vector Machine

**DOI:** 10.1371/journal.pone.0135709

**Published:** 2015-08-14

**Authors:** Fei Gao, Jingyuan Mei, Jinping Sun, Jun Wang, Erfu Yang, Amir Hussain

**Affiliations:** 1 School of Electronic and Information Engineering, Beihang University, Beijing, 100191, China; 2 Space Mechatronic Systems Technology Laboratory, Department of Design, Manufacture and Engineering Management, University of Strathclyde, Glasgow, G1 1XJ, United Kingdom; 3 Cognitive Signal-Image and Control Processing Research Laboratory, School of Natural Sciences, University of Stirling, Stirling, FK9 4LA, United Kingdom; Jiangnan University, CHINA

## Abstract

For current computational intelligence techniques, a major challenge is how to learn new concepts in changing environment. Traditional learning schemes could not adequately address this problem due to a lack of dynamic data selection mechanism. In this paper, inspired by human learning process, a novel classification algorithm based on incremental semi-supervised support vector machine (SVM) is proposed. Through the analysis of prediction confidence of samples and data distribution in a changing environment, a “soft-start” approach, a data selection mechanism and a data cleaning mechanism are designed, which complete the construction of our incremental semi-supervised learning system. Noticeably, with the ingenious design procedure of our proposed algorithm, the computation complexity is reduced effectively. In addition, for the possible appearance of some new labeled samples in the learning process, a detailed analysis is also carried out. The results show that our algorithm does not rely on the model of sample distribution, has an extremely low rate of introducing wrong semi-labeled samples and can effectively make use of the unlabeled samples to enrich the knowledge system of classifier and improve the accuracy rate. Moreover, our method also has outstanding generalization performance and the ability to overcome the concept drift in a changing environment.

## Introduction

With the arrival of the era of big data, today’s data has obvious characteristics of “4V” Volume, Velocity, Variety and Veracity, which not only puts forward higher requirements of storing data but also makes the tasks of data analysis and learning more difficult. Traditional machine learning methods are difficult to move forward because the constantly increasing data could not be stored infinitely and we also could not afford the high computational cost of re-learning all the data together when new data is acquired. Even the storage capacity and the computing speed could be improved continuously, however, these two points will of course speed up the coming of new data too, which means traditional classification strategies may still not keep up with the pace of data growth. In addition, new increasing data may have temporal correlation and change over time, which is the so-called “concept drift” [1, 2]. Without a forgetting mechanism, traditional methods are still hard to quickly and effectively adapt to changing environments so much so that an ideal learning may not be achieved.

The first paper about incremental learning can be traced back to 1962, when Coppock and Freund did a detailed study of biological learning process and got their paper published in the famous *Science* [3]. With the rapid developing of data classification, especially after SVM was proposed by Vapnik in 1995 [4], the incremental learning algorithm based on SVM has become a major research focus and is attracting the growing attention of many researchers. According to the purpose, the algorithms can be divided into two categories: 1) All the data has already been obtained while operating standard training process is infeasible due to its large amount so that the incremental learning techniques are introduced. 2) The new data is available at different time, for example: telephone records and weather conditions. To address this kind of problem, our learning process should become incremental. Meanwhile, according to the incremental learning approach, the algorithms can also be divided into two categories. For the first category, previous training set is used combined with new data as a new training set to re-train SVM. Redundant data is eliminated to achieve efficient incremental learning. In 1999, Syed et al. [5, 6] proposed a method that only support vectors are preserved and all other data is discarded after training. The method showed effectiveness in scalable data mining and enabled the incremental SVM to handle concept drift. On this basis, Mitra et al. [7] proposed a method for data condensation in large databases, in which the SVM is trained with the data in STORE, *n*_*m*_ misclassified samples and *n*_*c*_ correctly classified samples selected in GRABBAG with a resample technique and the support vectors are retained in STORE. The process is repeated in this method till the required accuracy is achieved on a testing set or GRABBAG is exhausted. In 2001, Domeniconi et al. [8] constructed incremental learning algorithms with SVM and four different techniques (ED, FP, EM, EM+E). In 2003, An et al. [9] proposed an incremental learning algorithm by considering the distance between the input pattern and the hyperplane. This method is widely used and expanded because of its efficient approach of improving the training speed, decreasing the memory requirement and guaranteeing the classification precision. In 2005, Erdem et al. [10, 11] found a new path to integrate the SVM into an ensemble framework using incremental Learn++ to address the catastrophic forgetting phenomenon. Cheng et al. [12] proposed an improved incremental training algorithm for support vector machines using active query in 2007, in which the k-means clustering algorithm is applied to select initial training samples, assign a weight to each sample in active query and develop a criterion to exclude non-informative training samples. For the second category, the SVM is modified to an online optimization algorithm to achieve the incremental process. In 2001, Cauwenberghs et al. [13] presented an incremental and decremental method using online recursive algorithm for training SVM, which had an excellent generalization performance and obtained the informative margin vectors and error vectors quickly and correctly. Then, in 2003, this method was extended by Cauwenberghs and Diehl [14] to a general framework for incremental learning, adaption and optimization. In 2006, Laskov et al. [15] made a deeper research on online incremental SVM learning scheme. They carried out a detailed analysis on the convergence and computational complexity of the algorithm, designed the new storage and the organization of operations and finally provided a fast, stable and robust implementation. In 2014, Cheng et al. [16] proposed a new incremental learning approach with margin-selective gradient descent learning to endow a fuzzy model with higher generalization ability and better expression of knowledge concept.

The introduction of incremental learning is a major breakthrough in the field of machine learning and more generally, computational intelligence. However, the applications of all these algorithms above require a premise that the labels of large amount of data acquired must be exactly known, which really means much human effort. So semi-supervised learning is expected could be combined with incremental learning to achieve more intelligent computation. From the study of human learning process, we found that incremental semi-supervised learning is feasible. As for a 6-month old infant, “dad” and “mum” these two words may be heard in many different cases. However, adults will not teach him/her clearly who is the “dad” and who is the “mum” each time. If we view that each time adults teach the infant to say “dad” and “mum” as labeled data and the sound of these two words in most cases as unlabeled data, the infant’s learning process is similar to an incremental semi-supervised learning process. Finally, the fact that he can distinguish these two words illustrates the feasibility of incremental semi-supervised mode and makes us believe the possibility of embedding this mode into computational intelligence.

Currently, there is only a small amount of literature [17, 18] about the structure of incremental semi-supervised learning algorithm based on the SVM. The research about this topic is hard and has many questions. The main difficulties can be concluded into two points. First, a 100% accuracy rate of the classes of semi-labeled samples could not be guaranteed by semi-supervised learning, which makes it easy to make a bigger mistake and lead to severe degradation in classifier performance in an incremental process. Second, strong model assumptions are made in many semi-supervised methods [19–21], which may be unable to match the problem structure of an incremental learning in a changing environment. In this paper, we propose a novel classification algorithm based on the incremental semi-supervised SVM. The first category of incremental learning approach is chosen because the second category approach with an online learning algorithm is required to know the exact labels of incoming samples in the initial stage, which is not conductive to the integration with semi-supervised theory. Meanwhile, for semi-supervised learning, self-training technique [22] is selected. The semi-labeled samples with high prediction confidence are collected, which is mainly according to the distances between each semi-labeled sample and all labeled samples and the distance between each semi-labeled sample and the optimal hyperplane of the SVM, without a strong model assumption of the real problem. In the initial stage of our algorithm, in order to avoid introducing some wrong semi-labeled samples into the training set, which may have a catastrophic influence on future learning, a “soft-start” approach is proposed to bring a relatively conservative attitude to the classifier and try to make the semi-labeled samples collection 100% correct. In the “soft-start” phase, the co-training technique [22] in semi-supervised theory is considered and an assistant classifier is introduced to help the main classifier. The distances between the introduced semi-labeled sample and all labeled samples are also restricted in a relatively tight range to ensure that our incremental semi-supervised algorithm has a smooth start. With the growth of main classifier, the assistant classifier will be abandoned and the distance restriction will be relaxed for collecting the semi-labeled samples faster. In addition, it is possible that some new sets of labeled samples may arise in the learning process. A detailed analysis about this condition is also carried out in this paper and the use of new labeled samples is maximized for the correction of some possible mistakes in the present classifier so that it will not be “lost in the forest” if a number of wrong semi-labeled samples are collected. Simulation results show that, for the two different purposes, our algorithm does not rely on the model of sample distribution and can effectively make use of new unlabeled samples to enrich the knowledge system of classifier and improve the accuracy rate. Moreover, the rate of introducing wrong semi-labeled samples is very low, which guarantees an excellent development of SVM in the learning process. Our method also has outstanding generalization performance and an ability to overcome the concept drift in a changing environment and reduces the amount of stored data and the cost of learning time.

This paper is structured as follows. The proposed algorithm based on the incremental semi-supervised SVM is described in Section 2. In Section 3, two sets of experiments are designed for two different purposes and the results are presented respectively. Section 4 presents our concluding remarks.

## Materials and Methods

The algorithm consists of four main parts: “soft-start”, incremental semi-supervised learning, data cleaning mechanism and new labeled samples’ learning. Details of the steps of our algorithm are explained in the following subsections.

### “Soft-start”

In the initial stage of the incremental semi-supervised learning, the knowledge structure of classifier is relatively weak since only a small number of labeled samples are available. Once some wrong semi-labeled samples are introduced into training set in this time it may hurt the progress of classifier in the future devastatingly. In order to ensure the credibility of classification results of the selected semi-labeled samples and achieve a stable “soft-start”, two classifiers (a main classifier and an assistant classifier) are used in our algorithm and the distances between the selected semi-labeled sample and all labeled samples are controlled strictly. Meanwhile, the distance between each semi-labeled sample and optimal hyperplane is another important reference index.

#### SVM classification

The two classifiers are trained with the labeled samples. To simplify the theoretical derivation process, the classification problem is assumed between two classes. We define:

$$l_{ij}=x_{i}•x_{j}$$ (1)

where *x*_*i*_(*i* = 1,2,⋯,*N*) and *x*_*j*_(*j* = 1,2,⋯,*N*) can be anyone of the labeled samples, *N* is the number of the labeled samples; *l*_*ij*_ is the dot product result. The kernel function for the main classifier is selected as Gaussian RBF (Radial Basis Function):

$$k_{1}(x_{i},x_{j})=\exp(-{\left\|x_{i}-x_{j}\right\|}^{2}/2\sigma^{2})=\exp\left[-(l_{ii}-2l_{ij}+l_{jj})/2\sigma^{2}\right]$$ (2)

While the kernel function for the assistant classifier is selected as polynomial function:

$$k_{2}(x_{i},x_{j})={\left(x_{i}•x_{j}+1\right)}^{p}={\left(l_{ij}+1\right)}^{p}$$ (3)

From (2) and (3), although an assistant classifier is added, the computation complexity is not increased much by calculating and recording *l*_*ij*_.

After training the two SVM classifiers, the classifier function is built:

$$f(x)=\mathrm{sgn}\{\sum_{i=1}^{N}a_{i}^{*}y_{i}k(x_{i},x^{new})+b^{*}\}$$ (4)

where *a*_*i*_ is the Lagrange multiplier corresponding to the *i*th sample and *C*≥*a*_*i*_≥0, *C* is the regularization parameter; *y*_*i*_ ∈ {+1,−1} is the class label associated to the *i*th labeled sample; *x* ^*new*^ denotes anyone of unlabeled samples; b* is a scalar and can be solved with any support vector *x*_*sv*_:

$$b^{*}=y_{i}-\sum_{x_{i}\in SV}a_{i}^{*}y_{i}k(x_{i},x_{sv})$$ (5)

In (4), we let $d_{1}(x^{new})=\left|\sum_{i=1}^{N}a_{i}^{*}y_{i}k_{1}(x_{i},x^{new})+b^{*}\right|$ denote the distance between sample *x* ^*new*^ and the separation hyperplane of main classifier. The prediction confidence of *x* ^*new*^ may be higher if *d*_1_(*x* ^*new*^) is larger. Furthermore, when the density of new incoming unlabeled samples near the optimal hyperplane is very high, it often predicts the distribution of original data is different with the distribution of new unlabeled data. In other words, the “concept drift” occurs. If the density exceeds acceptable range, classification results given by the current main classifier may carry low confidence. As *d*_1_(*x*_*sv*_) = 1, we let *d*_*threshold*_ = 0.1; *count*(*d*_1_(*x* ^*new*^)<*d*_*threshold*_) denote the number of new incoming unlabeled samples with *d*_1_(*x* ^*new*^)<*d*_*threshold*_; *N* ^*new*^ denotes the total number of new incoming unlabeled samples; *p* ∈ [0.8, 0.9] is a scale factor. If

$$count(d_{1}(x^{new})<d_{threshold})/N^{new}\leq p$$ (6)

it means the proportion of unlabeled samples in the vicinity of the hyperplane does not exceed the scale factor *p* so that this batch of data is suitable for learning by the main classifier. Otherwise, the main classifier will only announce the classification results without learning the new data.

#### Distance between semi-labeled sample and hyperplane

**Fig 1:**
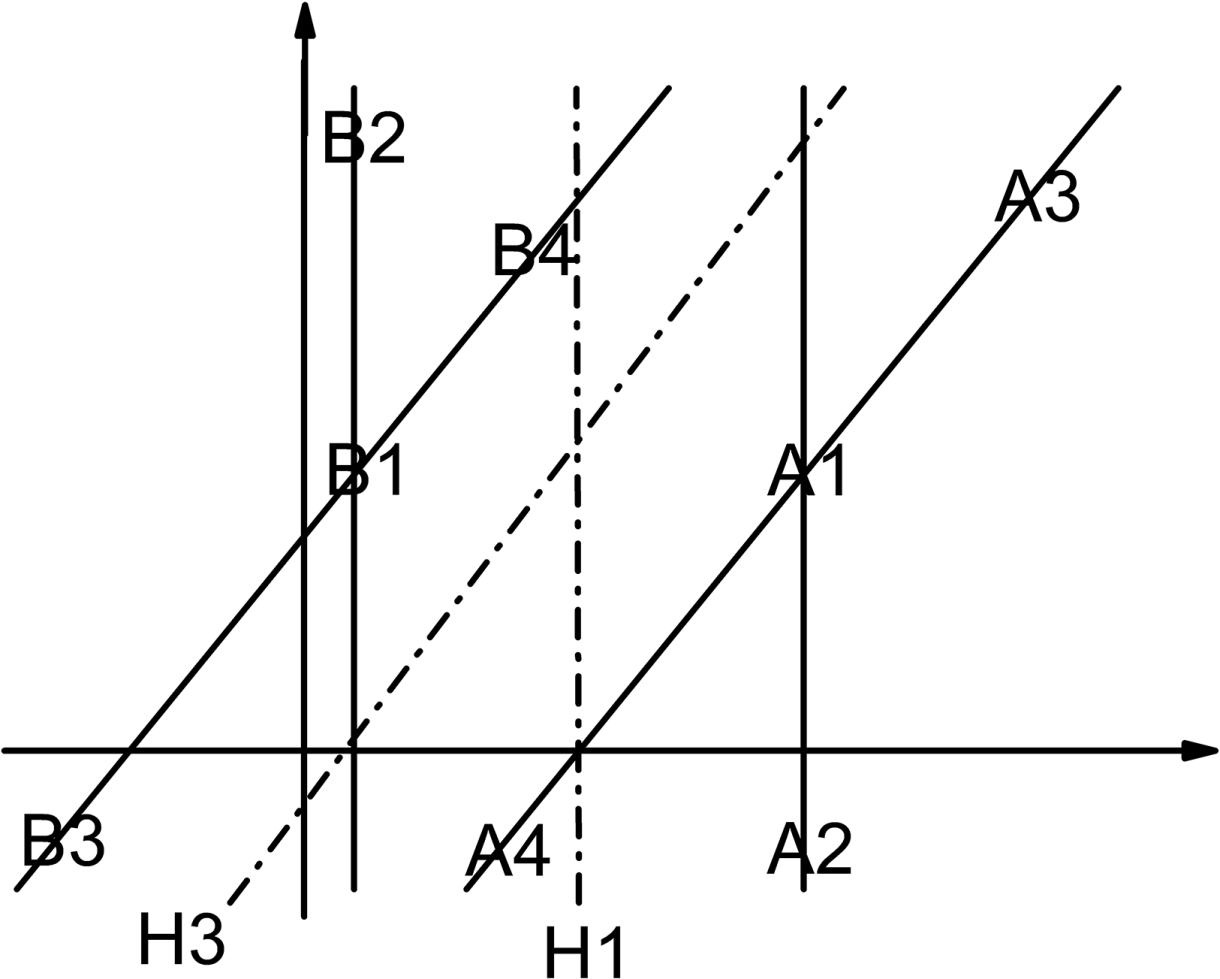
Non-support vectors become support vectors (H1 is the initial hyperplane. H3 is the final hyperplane. A1, A2, A3, A4, B1, B2, B3 and B4 are samples).

For the batch which satisfies (6), the distance between the semi-labeled sample and the hyperplane will be considered first. Let *x* ^*c_new*^(*c* = 1, 2) denote anyone of the new semi-labeled samples which are divided into class *c* by the main classifier. For all the samples with *d*_1_(*x* ^*c_new*^)<1, a threshold *d* ^*c_new*^ is set for selecting the samples with a higher prediction confidence in class *c*. We denote that *N* ^*c_new*^ is the number of samples which satisfy *d*_1_(*x* ^*c_new*^)<1 in class *c*, thus

$$d^{c\_new}=\max(d_{1}(x_{i}^{c\_new}))\sum_{i=1}^{N^{c\_new}}d_{1}(x_{i}^{c\_new})/N^{c\_new}$$ (7)

where max represents the operation to take the maximum value. If *d* ^*c_new*^ ≤ *d*_1_(*x* ^*c_new*^)<1, *x* ^*c_new*^ is considered as a candidate semi-labeled sample in the margin band to be introduced into the training set. For the samples which satisfy *d*_1_(*x* ^*c_new*^)≥1, all of them will also be added into the candidate sample set. The reasons for this point are: First, *d*_1_(*x* ^*c_new*^)≥1 means that *x* ^*c_new*^ carries a very high credibility. Second, as the distances between each semi-labeled sample and all the labeled samples will still be computed, collecting more samples with *d*_1_(*x* ^*c_new*^)≥1 will help the main classifier to obtain the semi-labeled samples more quickly and expand the space of distribution of labeled samples. Third, new samples have the most information of current concept so that collecting more may be beneficial to solve “concept drift” problem. As the hyperplane of the SVM is only relevant to support vectors, in a lot of literature [5–7, 23] the samples with *d*_1_(*x* ^*c_new*^)>1 are abandoned directly, however in our research we find that previous non-support vectors are quite possible to become support vectors when the number of training samples increases or concept drifts. The schematic drawing is shown in Fig 1. We assume: the initial hyperplane is H1; A1 and A2 are the initial support vectors for class A; B1 and B2 are the initial support vectors for class B; A3 and B3 are the initial non-support vectors. After introducing A4 and B4, the hyperplane becomes H3 and A3 and B3 become the support vectors. Fig 1 effectively illustrates that preserving non-support vectors is not insignificant.

**Fig 2:**
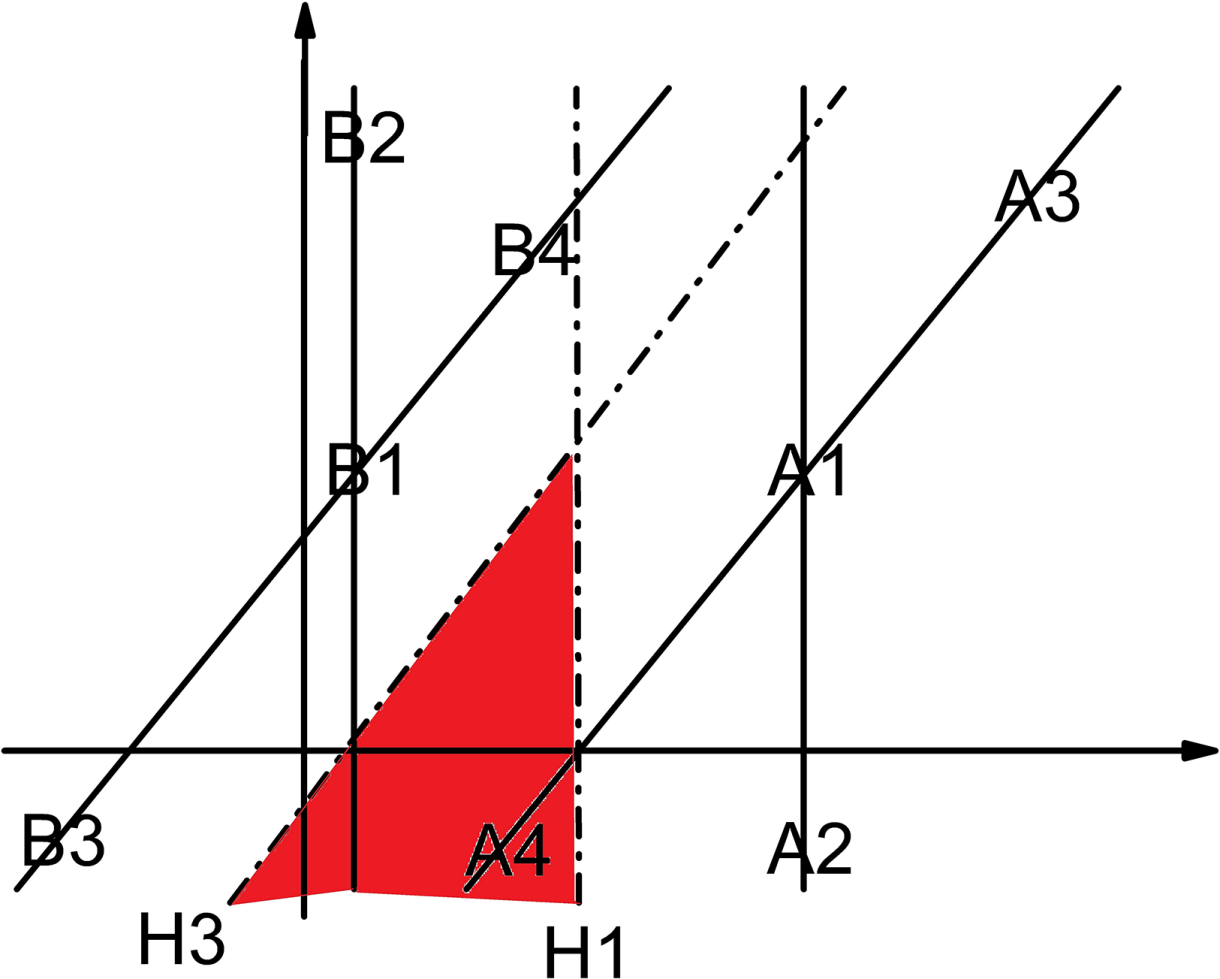
The distance between the semi-labeled sample and the hyperplane is far but the classification result is still wrong (H1 is the initial hyperplane. H3 is the final hyperplane. A1, A2, A3, A4, B1, B2, B3 and B4 are samples).

A lot of literature [24–27] on semi-supervised learning depend only on the distance between each semi-labeled sample and the hyperplane to determine the credibility of this semi-labeled sample, which we think is one-sided. For example, sometimes the hyperplane may be generated by few training samples, which could not reflect the actual distribution of different classes of samples. In this condition, it is possible that the classification result is wrong although the sample is far away from the hyperplane. The schematic illustration is shown in Fig 2. All the assumptions are the same with Fig 1. For the hyperplane H3 to truly reflect the distribution relationship of class A and class B, the unlabeled samples in the red area and its unlimited extension should be divided into class A, however they will be divided into class B by the hyperplane H1 even if they are infinitely far away from H1. Fig 2 effectively illustrates that the classification result is not necessarily correct even the distance between the semi-labeled sample and the hyperplane is far enough.

#### Distances between each semi-labeled sample and labeled samples

In this paper, the distances between each semi-labeled sample and all the labeled samples in this class are considered as important indicators for the prediction confidence of this semi-labeled sample. As we know from Fig 2, although the semi-labeled samples in the red area are far away from H1, they are not close enough to B1, B2 or B3, which increases the uncertainty of their classification results. For the main classifier, after projecting the labeled samples into the Reproducing Kernel Hilbert Space *F* by a nonlinear mapping *ϕ*:*x* ∈ *R* ^*n*^ → *ϕ*(*x*) ∈ *F*, we let $x_{i}^{c}(i=1,2,\cdots ,N^{c})$ denote the *i*th labeled sample in class *c* in the original space; $\phi_{i}^{c}$ denotes the projection of $x_{i}^{c}$ in space *F*; *N* ^*c*^ denotes the total number of labeled samples in class *c*. In Euclidean space, the distance between any two labeled samples in class *c* in space *F* can be written as:

$$\left|\phi_{i}^{c}-\phi_{j}^{c}\right|=\sqrt{{\left(\phi_{i}^{c}\right)}^{2}-2(\phi_{i}^{c}•\phi_{j}^{c})+{\left(\phi_{j}^{c}\right)}^{2}}=\sqrt{k_{1}(x_{i},x_{i})-2k_{1}(x_{i},x_{j})+k_{1}(x_{j},x_{j})}$$ (8)

As *k*_1_(*x*_*i*_,*x*_*i*_) = *k*_1_(*x*_*j*_,*x*_*j*_) = 1, (2), (8) can be simplified as:

$$\left|\phi_{i}^{c}-\phi_{j}^{c}\right|=\sqrt{2-2k_{1}(x_{i},x_{j})}=\sqrt{2-2\exp\left[-(l_{ii}-2l_{ij}+l_{jj})/2\sigma^{2}\right]}$$ (9)

The distance $\left|\phi_{i}^{c}-\phi_{j}^{c}\right|$ can also be calculated on the basis of *l*_*ij*_, which greatly reduces the computational complexity of our proposed algorithm. For any semi-labeled sample *x* ^*c_new*^, the distance between *x* ^*c_new*^ and each labeled sample in class *c* in space *F* is

$$\left|\phi (x^{c\_new})-\phi_{i}^{c}\right|=\sqrt{2-2k_{1}(x_{i},x^{c\_new})}$$ (10)

where the computation of *k*_1_(*x*_*i*_,*x* ^*c_new*^) has already been done in (4). In this paper, we prefer to collect the semi-labeled samples which are not far from the labeled samples so that *x* ^*c_new*^ will be a candidate

$$\underset{i=1,2,\cdots ,N^{c}}{\min}(\left|\phi (x^{c\_new})-\phi_{i}^{c}\right|)\leq \underset{\begin{array}{c} i,j=1,2,\cdots ,N^{c}\\ i\neq j \end{array}}{\min}(\left|\phi_{i}^{c}-\phi_{j}^{c}\right|)$$ (11)

where min represents the minimum-value operator. Eq (11) shows that the minimum distance between a candidate semi-labeled sample and each labeled sample of its class should not be greater than the minimum distance between any two labeled samples in this class. The possibility of semi-labeled samples in isolation is decreased with this indicator so as to enhance the credibility.

So far, in the “soft-start” phase, for any new incoming semi-labeled sample *x* ^*c_new*^, if

$$\left\{ \begin{array}{l} \text{Main classifier result}=\text{Assistant classifier result}\\ d_{1}(x^{c\_new})\geq d^{c\_new}\\ \underset{i=1,2,\cdots ,N^{c}}{\min}(\left|\phi (x^{c\_new})-\phi_{i}^{c}\right|)\leq \underset{\begin{array}{c} i,j=1,2,\cdots ,N^{c}\\ i\neq j \end{array}}{\min}(\left|\phi_{i}^{c}-\phi_{j}^{c}\right|) \end{array}\right.$$ (12)

*x* ^*c_new*^ will be put into the training set of the main classifier as a labeled sample, while for the assistant classifier it only provides the classification results without collecting any new samples.

Since our algorithm is going to handle the new data continuously, the previous data is not read again once it is abandoned. Thus it is faced with the problem that it is difficult for the main classifier to obtain more new samples when $\underset{\begin{array}{c} i,j=1,2,\cdots ,N^{c}\\ i\neq j \end{array}}{\min}(\left|\phi_{i}^{c}-\phi_{j}^{c}\right|)$ is too small. In order to address this problem, two buffers are created to save the samples which satisfy (13)

$$\underset{\begin{array}{c} i,j=1,2,\cdots ,N^{c}\\ i\neq j \end{array}}{\min}(\left|\phi_{i}^{c}-\phi_{j}^{c}\right|)<\underset{i=1,2,\cdots ,N^{c}}{\min}(\left|\phi (x^{c\_new})-\phi_{i}^{c}\right|)\leq \underset{\begin{array}{c} i=1,2,\cdots ,N^{c}\\ i\neq j \end{array}}{\max}\left[\underset{j=1,2,\cdots ,N^{c}}{\min}(\left|\phi_{i}^{c}-\phi_{j}^{c}\right|)\right]$$ (13)

For the first batch of data, the semi-labeled samples which satisfy (13) are saved in the first buffer while the semi-labeled samples in the second batch which satisfy (13) are saved in the second buffer. When the third batch arrives, it is sent to the classifiers together with the samples in the first buffer. The above computation is repeated and the samples that satisfy (13) are saved in the first buffer again. For the fourth batch, it is processed with the data in the second buffer. The same work is done recursively for every new batch in the future.

**Fig 3:**
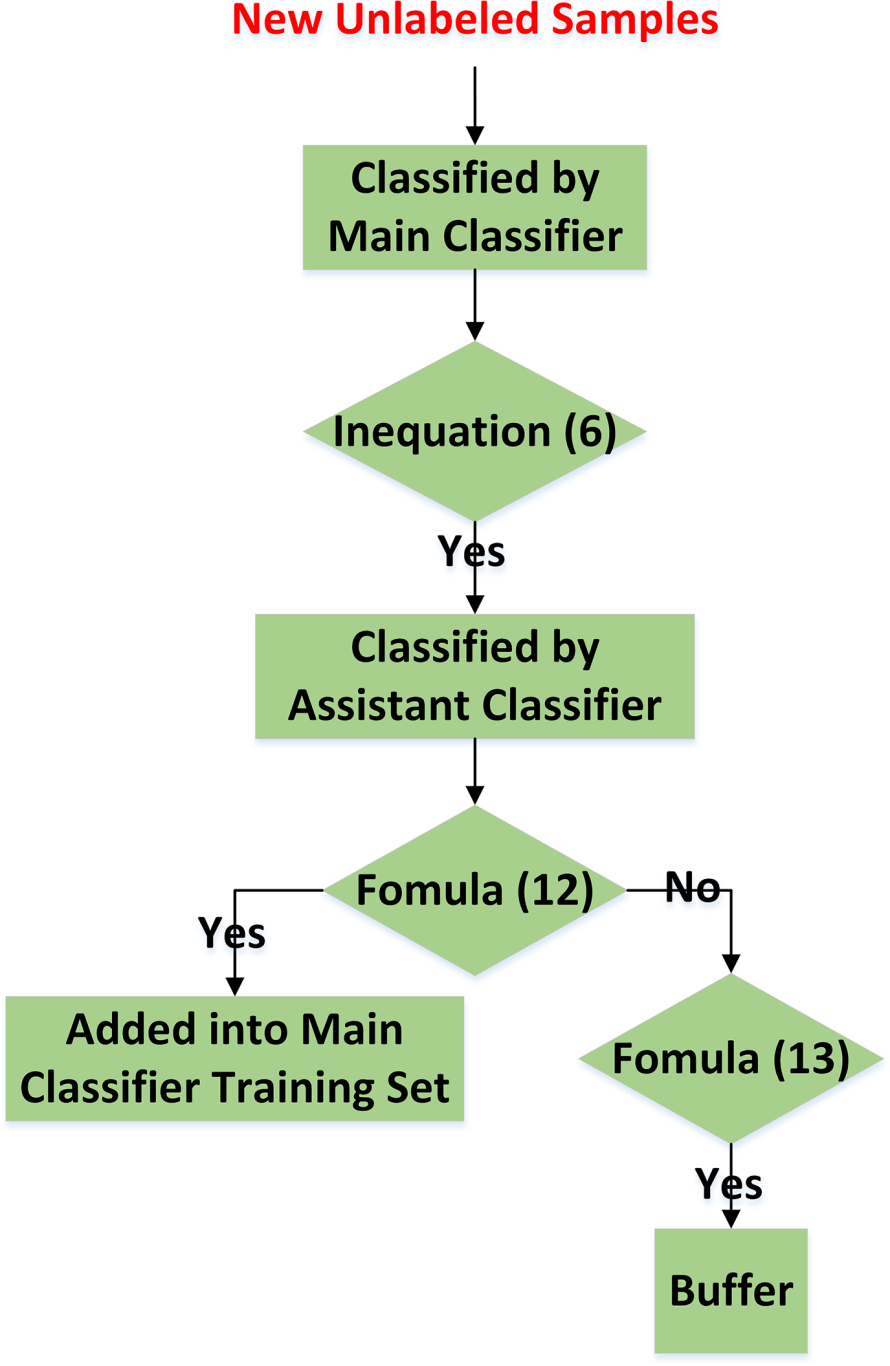
Flow-chart of “soft-start.”

The procedure of “soft-start” is illustrated in Fig 3.

### Incremental semi-supervised learning

After the “soft-start” phase, as a number of the semi-labeled samples with high confidence have been absorbed into the training set of the main classifier, the knowledge structure of the main classifier become more abundant. At this point, we can relax the conditions of introducing the semi-labeled samples and try to achieve a quicker introduction. First, the assistant classifier is abandoned. With further learning, as no new samples are collected by the assistant classifier, it could not solve the “concept drift” problem in a changing environment and may have interference on the classification decision. Second, the restriction of (11) and (13) is relaxed. With more and more new samples being introduced into the training set, the value of $\underset{\begin{array}{c} i,j=1,2,\cdots ,N^{c}\\ i\neq j \end{array}}{\min}(\left|\phi_{i}^{c}-\phi_{j}^{c}\right|)$ and $\underset{\begin{array}{c} i=1,2,\cdots ,N^{c}\\ i\neq j \end{array}}{\max}\left[\underset{j=1,2,\cdots ,N^{c}}{\min}(\left|\phi_{i}^{c}-\phi_{j}^{c}\right|)\right]$ will inevitably become smaller, which results in it being extremely difficult introducing work. In this regard, in order to ensure the speed of learning new data, (11) is changed into

$$\underset{i=1,2,\cdots ,N^{c}}{\min}(\left|\phi (x^{c\_new})-\phi_{i}^{c}\right|)\leq \rho •\underset{\begin{array}{c} i=1,2,\cdots ,N^{c}\\ i\neq j \end{array}}{\text{mean}}\left[\underset{j=1,2,\cdots ,N^{c}}{\min}(\left|\phi_{i}^{c}-\phi_{j}^{c}\right|)\right]$$ (14)

in which mean represents the operator to take average value; *ρ* ≥ 1 is the distance control constant. Meanwhile, (12) is changed into

$$\left\{ \begin{array}{l} d_{1}(x^{c\_new})\geq d^{c\_new}\\ \underset{i=1,2,\cdots ,N^{c}}{\min}(\left|\phi (x^{c\_new})-\phi_{i}^{c}\right|)\leq \rho •\underset{\begin{array}{c} i=1,2,\cdots ,N^{c}\\ i\neq j \end{array}}{\text{mean}}\left[\underset{j=1,2,\cdots ,N^{c}}{\min}(\left|\phi_{i}^{c}-\phi_{j}^{c}\right|)\right] \end{array}\right.$$ (15)

(13) is also changed into

$$\rho •\underset{\begin{array}{c} i=1,2,\cdots ,N^{c}\\ i\neq j \end{array}}{\text{mean}}\left[\underset{j=1,2,\cdots ,N^{c}}{\min}(\left|\phi_{i}^{c}-\phi_{j}^{c}\right|)\right]<\underset{i=1,2,\cdots ,N^{c}}{\min}(\left|\phi (x^{c\_new})-\phi_{i}^{c}\right|)\leq \rho •\underset{\begin{array}{c} i=1,2,\cdots ,N^{c}\\ i\neq j \end{array}}{\max}\left[\underset{j=1,2,\cdots ,N^{c}}{\min}(\left|\phi_{i}^{c}-\phi_{j}^{c}\right|)\right]$$ (16)

**Fig 4:**
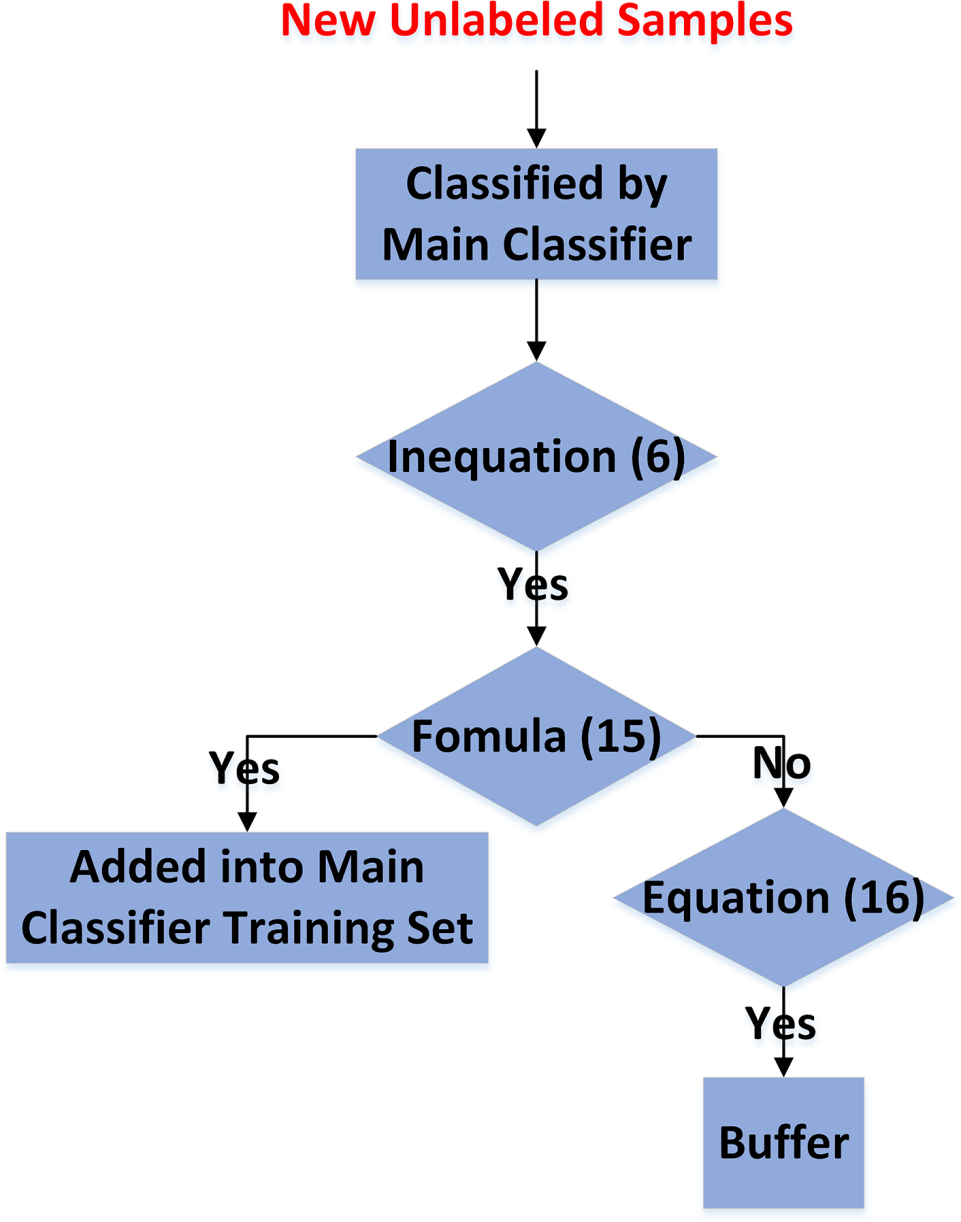
Flow-chart of incremental semi-supervised learning.

The procedure of incremental semi-supervised learning is illustrated in Fig 4.

### New labeled samples’ learning

In the learning process, some new labeled samples may also be obtained, which are precious resources for incremental semi-supervised learning. We hope that new labeled samples could be used not only to enhance our knowledge system but also to estimate the performance of main classifier and help it recover from the errors that may exist. In this paper, they are classified by (4) firstly and the classification results are compared with their real labels. If some of the results are wrong, we analyze that there are two main reasons: First, the misclassified samples may be corrupted by some noise or have some special attributes that the current classifier is unable to output the correct results. Second, some misclassified semi-labeled samples are introduced into the training set in the previous learning process, which causes a certain degree of classification confusion. Apparently, the impact of the second reason on the classifier is fatal, which is likely to lead to consecutive mistakes in the future. Thus, in order to exclude the possibility of introducing wrong semi-labeled samples, our method chooses to clear all the previous semi-label samples in the circle with the center at every misclassified new labeled sample and the radius equals to the value in Eq (14) as:

$$r=\rho •\underset{\begin{array}{c} i=1,2,\cdots ,N^{c}\\ i\neq j \end{array}}{\text{mean}}\left[\underset{j=1,2,\cdots ,N^{c}}{\min}(\left|\phi_{i}^{c}-\phi_{j}^{c}\right|)\right]$$ (17)

In addition, for the correctly classified labeled samples they are saved directly.

**Fig 5:**
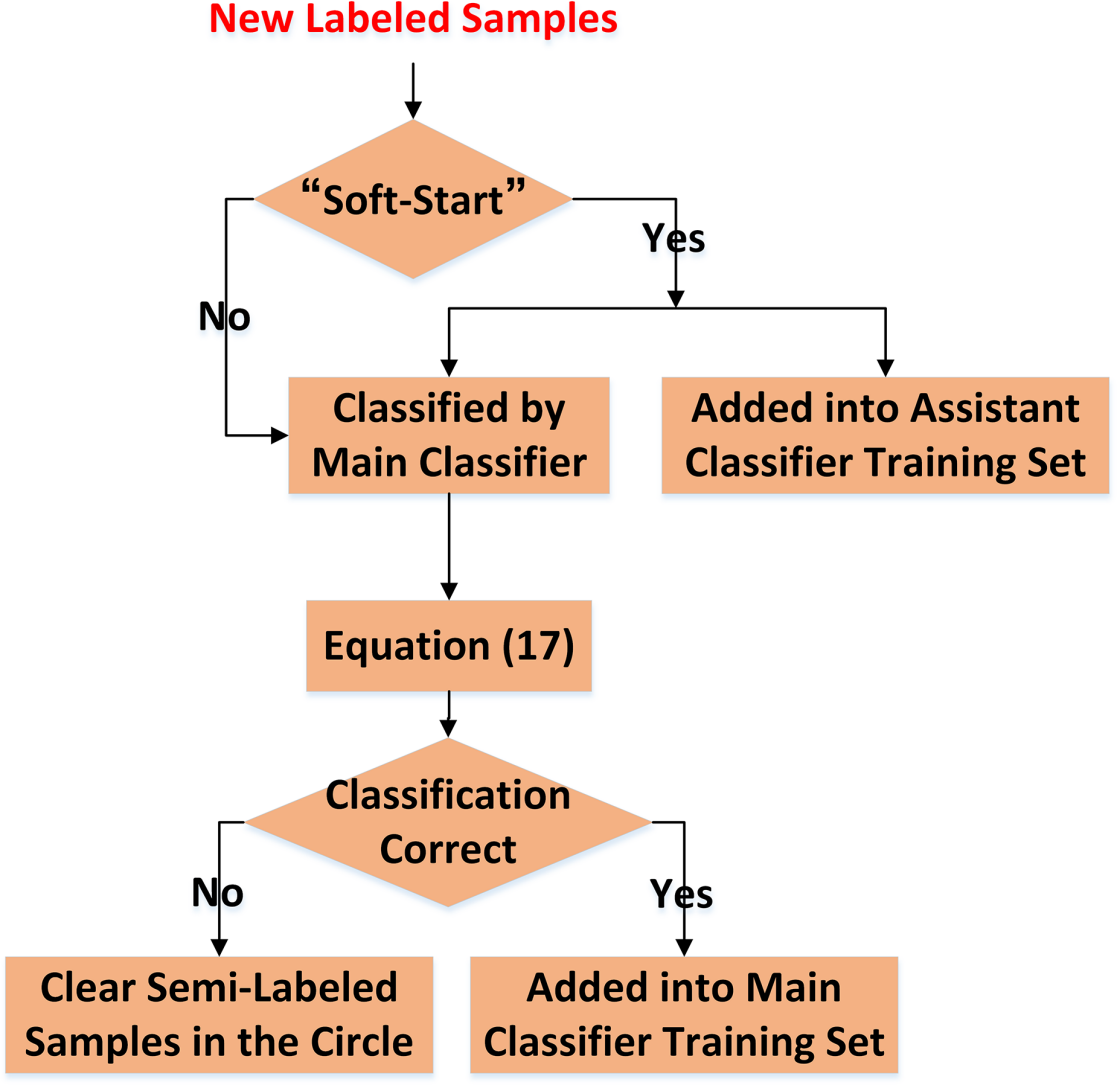
Flow-chart of new labeled samples’ learning.

The procedure of new labeled samples’ learning is illustrated in Fig 5.

### Data cleaning mechanism

A further study discovers that the number of non-support vectors gradually rises, which means more storage space and longer training time. Therefore, a data cleaning mechanism is proposed. In order to make the classifier have the best performance of the distribution of current data, our mechanism does not clean up the latest batch of data but only the previous data. When the length of the training time or the amount of data storage reaches the upper threshold, for all the samples in the previous training set satisfying $d_{1}(x_{i}^{c})>1$, we set

$$d^{c}=N^{c\_old}\max(d_{1}(x_{i}^{c}))/\sum_{i=1}^{N^{c\_old}}d_{1}(x_{i}^{c})$$ (18)

where *N* ^*c_old*^ is the number of samples in the previous training set satisfying $d_{1}(x_{i}^{c})>1$. $x_{i}^{c}$ will be discarded if $d_{1}(x_{i}^{c})>d^{c}$. As $x_{i}^{c}$ is one of the training samples, we can assume that it is the *k*th(1 ≤ *k* ≤ *N*) sample in the training set as $x_{i}^{c}=x_{k}$. According to (4) and (2), $d_{1}(x_{i}^{c})$ can be written as:

$$d_{1}(x_{i}^{c})=\left|\sum_{j=1}^{N}a_{j}^{*}y_{j}k_{1}(x_{j},x_{k})+b^{*}\right|=\left|\sum_{j=1}^{N}a_{j}^{*}y_{j}\exp\left[-(l_{jj}-2l_{jk}+l_{kk})/2\sigma^{2}\right]+b^{*}\right|$$ (19)

It is obvious that the data cleaning mechanism is based on the computation of *l* as well. Since the data cleaning work is carried out by the class label, the class with more data is cleaned up first in order to alleviate the problem of unbalanced classes.

**Fig 6:**
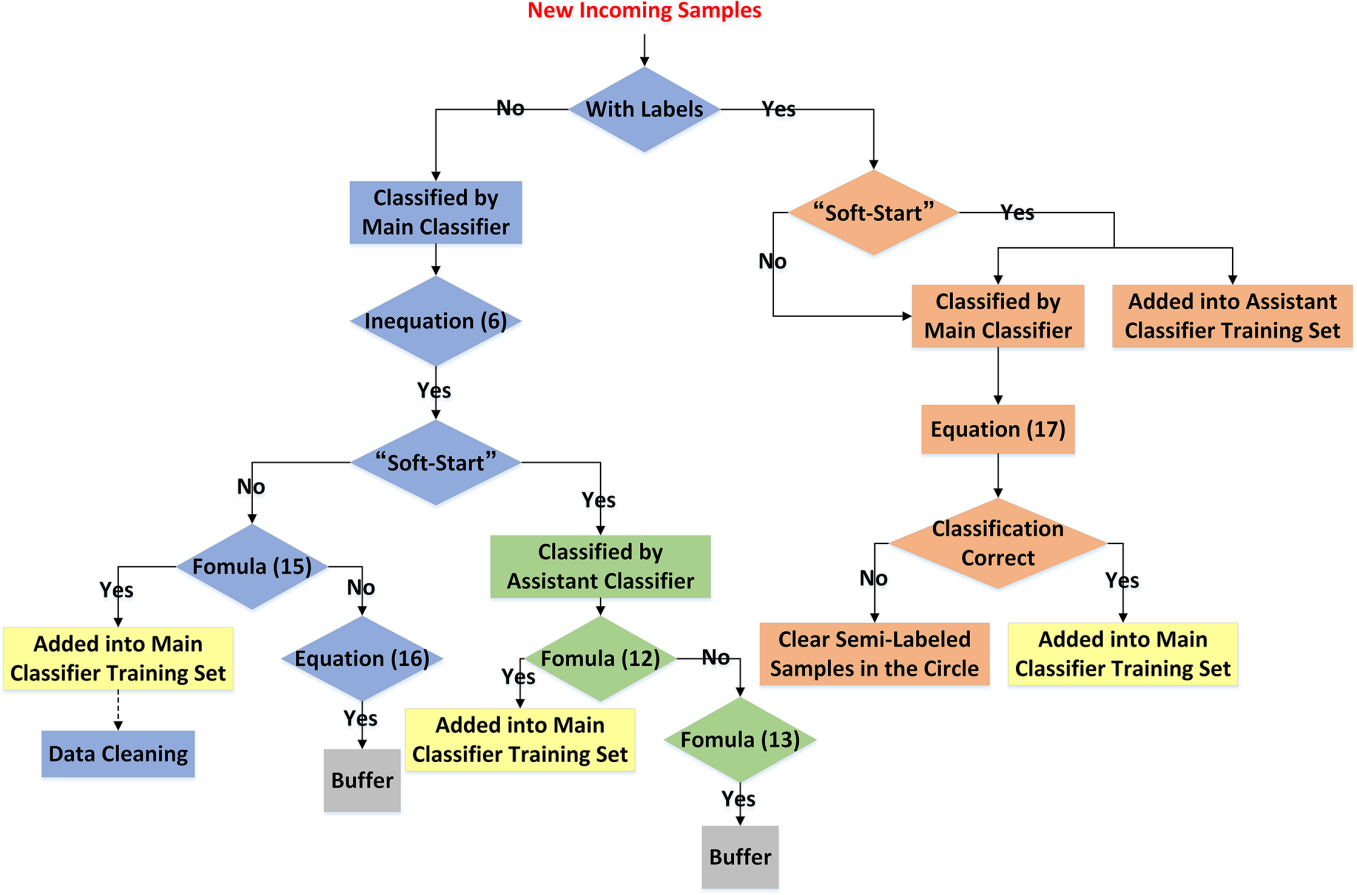
Flow-chart of our classification algorithm based on incremental semi-supervised SVM.

At this point, all the procedure of the novel classification algorithm based on the incremental semi-supervised SVM proposed in this paper is completed. The whole procedure is also illustrated in Fig 6. Moreover, our proposed algorithm can be easily extended into multiple classification applications.

## Results and Discussion

In this section, two sets of experiments for the two different purposes of incremental learning were designed respectively. In order to validate the generalization ability of our algorithm, MSTAR database [28] and FACE database [12] from MIT (data in S1 Dataset) were used in our two experiments respectively. All the experiments were conducted on an Intel Core2 E8400 3.0GHz CPU, running the Windows 7 operating system, with programs written in Matlab.

### MSTAR database

In this experiment, BMP2 (232 sn-c21 s (data in S2 Dataset), 191 sn-9563 s (data in S3 Dataset), 191 sn-9566 s (data in S4 Dataset)) and T72 (232 sn-132 s (data in S5 Dataset), 191 sn-812 s (data in S6 Dataset), 191 sn-s7 s (data in S7 Dataset)) at 17° depression angle were chose as the sample set. All the 1228 vehicles above were mixed and divided randomly as follows: 61 BMP2 s and 61 T72 s were grouped as the initial labeled training set. 53 BMP2 s and 53 T72 s were grouped as a batch of new labeled samples which appeared during the learning process. 50 BMP2 s and 50 T72 s were grouped as a batch of the unlabeled samples and there were 10 batches in all. Meanwhile, 191 BMP2sn-c21 s at 15° depression angle (data in S8 Dataset) were chose as the testing set in this experiment, which could help us to follow the accuracy rate of the main classifier after learning each batch of data.

The first three steps were set as the “soft-start” phase. For the kernel function in the main classifier, we set 1 / 2*σ* ^2^ = 0.6. For the kernel function in the assistant classifier, we set *p* = 2. Before the classification, it was necessary to normalize the amplitude of each sample. The normalization formula was given by:

$$x_{Normalized}=x/{\left\|x\right\|}_{2}$$ (20)

where **x** was the vector representation of each sample; **x**_*Normalized*_ was the vector representation of the normalized **x**.

**Fig 7:**
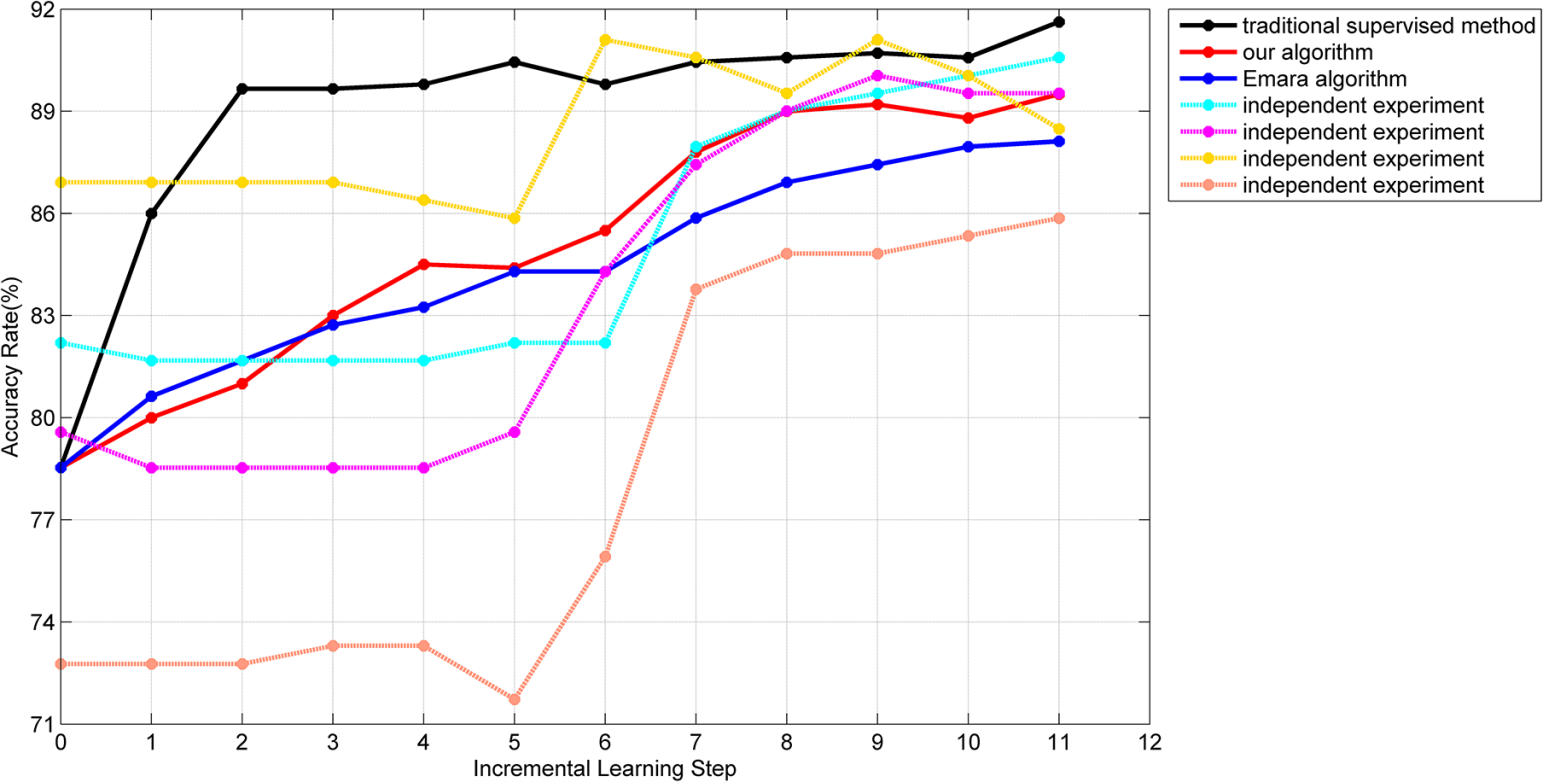
The accuracy rate of the testing set with different algorithms.

As each series of vehicles had three different sub-series and each batch of data was grouped randomly, it made the correlation between any two batches weak and the distribution of each batch had a great deal of variability, which meant our algorithm learnt in a changing environment. The experiment was conducted multiple times and the accuracy rates of different algorithms were plotted in Fig 7.

In Fig 7, the abscissa represents the incremental learning process, where 0 represents the initial training with the labeled samples, the 7^th^ batch of data is the new labeled sample set and all other batches are the unlabeled sample sets. The ordinate represents the accuracy rate of the main classifier on the testing set after the completion of classification and learning of each batch of data. 4 dashed lines represent the processes of 4 independent experiments with our algorithm. The average processes of 100 independent experiments of our algorithm, Emara’s algorithm [18] and the traditional supervised learning method are represented with red, blue and black solid lines respectively.

Since the initial training samples are randomly selected, there is a big change in the accuracy rate in the original stage, which may also have an influence on future learning. However, the 4 dashed lines show that our proposed incremental semi-supervised algorithm based on the SVM makes the accuracy rate have different degrees of improvement, which proves that our algorithm can effectively enrich the knowledge structure of the SVM with unlabeled samples. Compared with the algorithm in [18], although there is a slower improvement in the “soft-start” phase, an obviously better learning effect is presented by our method. The main reasons are summarized. First, in our algorithm, a stricter credibility test is processed for each new semi-labeled sample, which leads to lower possibility of misclassification. Second, in Emara’s algorithm, the labels of semi-labeled samples may be removed after each new sample arriving, which may cause training set instability and limit the growth of the accuracy rate. The comparison between red and black solid lines also shows that the accuracy rate of our incremental semi-supervised method is close to the traditional supervised method and illustrates that our method is applicable to the first purpose of incremental learning. In addition, in the changing environment, the accuracy rate of our method does not drop significantly and the number of introduced misclassified semi-labeled samples is precious few (only 1 in the 11^th^ step of the yellow curve), which demonstrates the stability of the proposed “soft-start”, the correctness of our selection of semi-labeled samples and our adaptability of “concept drift”.

Here we need to emphasize that the rise of accuracy rate of our algorithm, as a semi-supervised algorithm, should be mainly based on the collection of the unlabeled samples. The reason to consider the emergence of the new labeled samples is that we hope our algorithm could have a better generalization performance of new data and be closer to the practical applications. In the first 6 batches, a rise takes place in the yellow, purple and orange curves while the cyan curve is relatively stable, which is determined by the number of introduced semi-labeled samples. For the yellow, purple and orange curves, each of them obtains near 145 semi-labeled samples while the number for the cyan curve is only 86, which makes it hard to have a remarkable advance. For the last 4 batches, with the relaxation of indicator in (14), the speed of obtaining new samples accelerates and the accuracy rate of each curve maintains at a high level and still has a different range of improvement. Finally, the average number of training samples in our method will exceed 800. The analysis demonstrates that our proposed algorithm has a good ability of the incremental semi-supervised learning.

**Fig 8:**
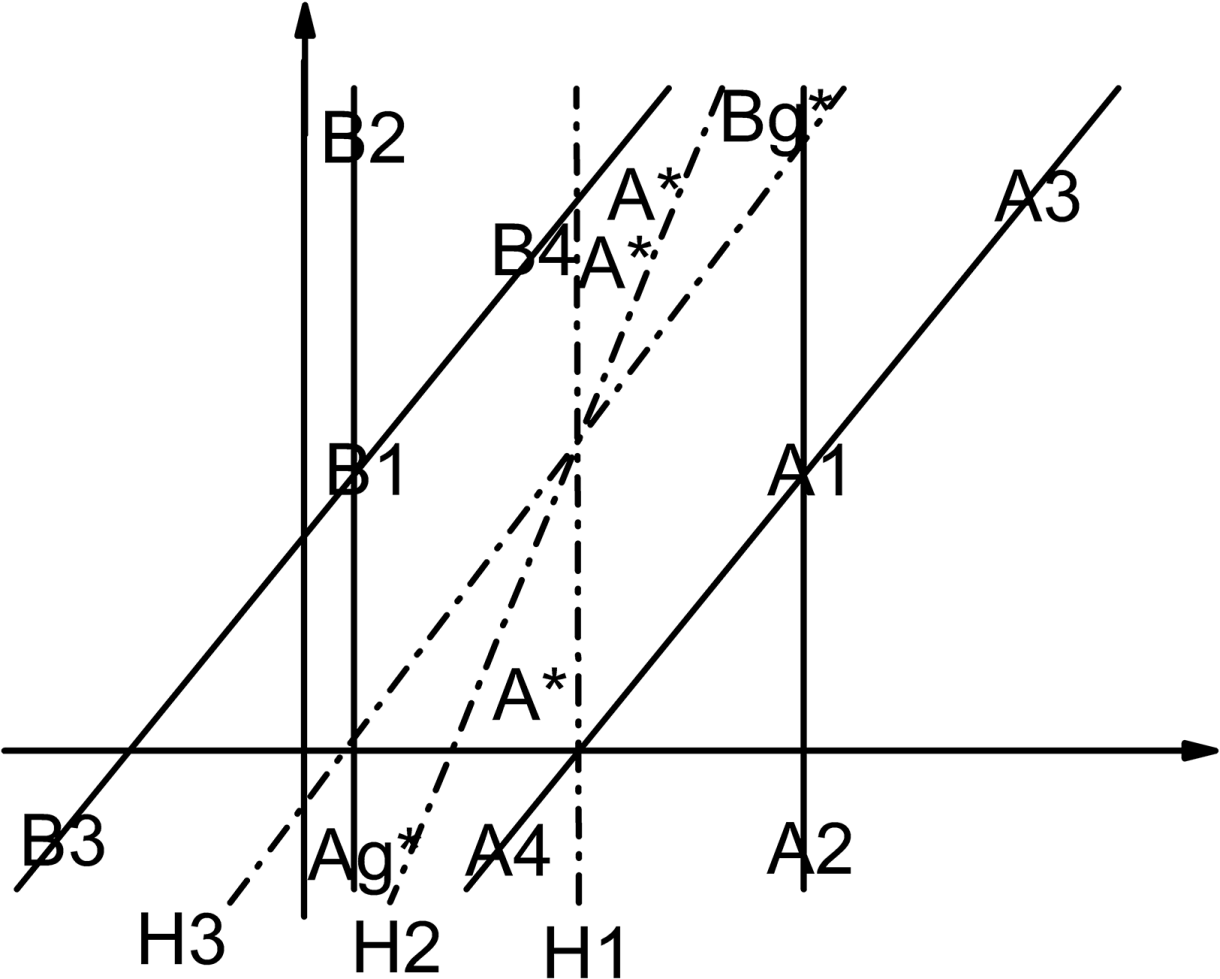
All the semi-labeled are introduced correctly but the accuracy rate declines (H1 is the initial hyperplane, H3 is the final hyperplane and H2 is the hyperplane at a certain moment in the learning process. A1, A2, A3, A4, B1, B2, B3 and B4 are samples. A* represents a testing sample in class A. Ag* and Bg* represent a group of testing samples in class A and class B, respectively).

For the drop phenomenon in each curve, it is not a signal that some mislabeled samples are introduced into the training set. It is possible that all the introduced samples are correct but the accuracy rate still declines. In this regard, a schematic illustration is presented in Fig 8 to explain this phenomenon.

For H1, the upper two A* s are classified correctly while the lower A*, Ag* and Bg* are classified mistakenly. For H3, the upper two A* s are classified mistakenly while the lower A*, Ag* and Bg* are classified correctly, which means the accuracy rate of H3 improves significantly relative to H1. However, for the hyperplane H2 at an intermediate moment, only the lower A* is classified correctly, which results in a moment that all the semi-labeled are introduced correctly but the accuracy rate is worse than the initial stage.

### FACE database

This experiment was focused on the second purpose of incremental learning and was designed to validate the ability of our algorithm to discriminate and learnt the new data. In order to reflect the effectiveness of solving “concept drift”, we made a choice of the first 50 samples in face training set and the first 50 samples in class B1 in non-face training set as our initial training set, and also other 340 samples in the face training set and the first 340 samples in class B5 in non-face training set were selected as the unlabeled incremental sets. The 680 incremental samples were randomly divided into 10 batches. Each batch had 34 face samples and 34 non-face samples. Since there was a large difference between class B1 and class B5, the learning process was analyzed through the accuracy rate of each new batch. For fully considering some extreme cases, the “concept drift” occurred directly from the first batch, which could better validate the ability of our algorithm to learn in the changing environment. Some non-support vectors were also abandoned with our data cleaning mechanism after learning the 7^th^ batch of data.

**Fig 9:**
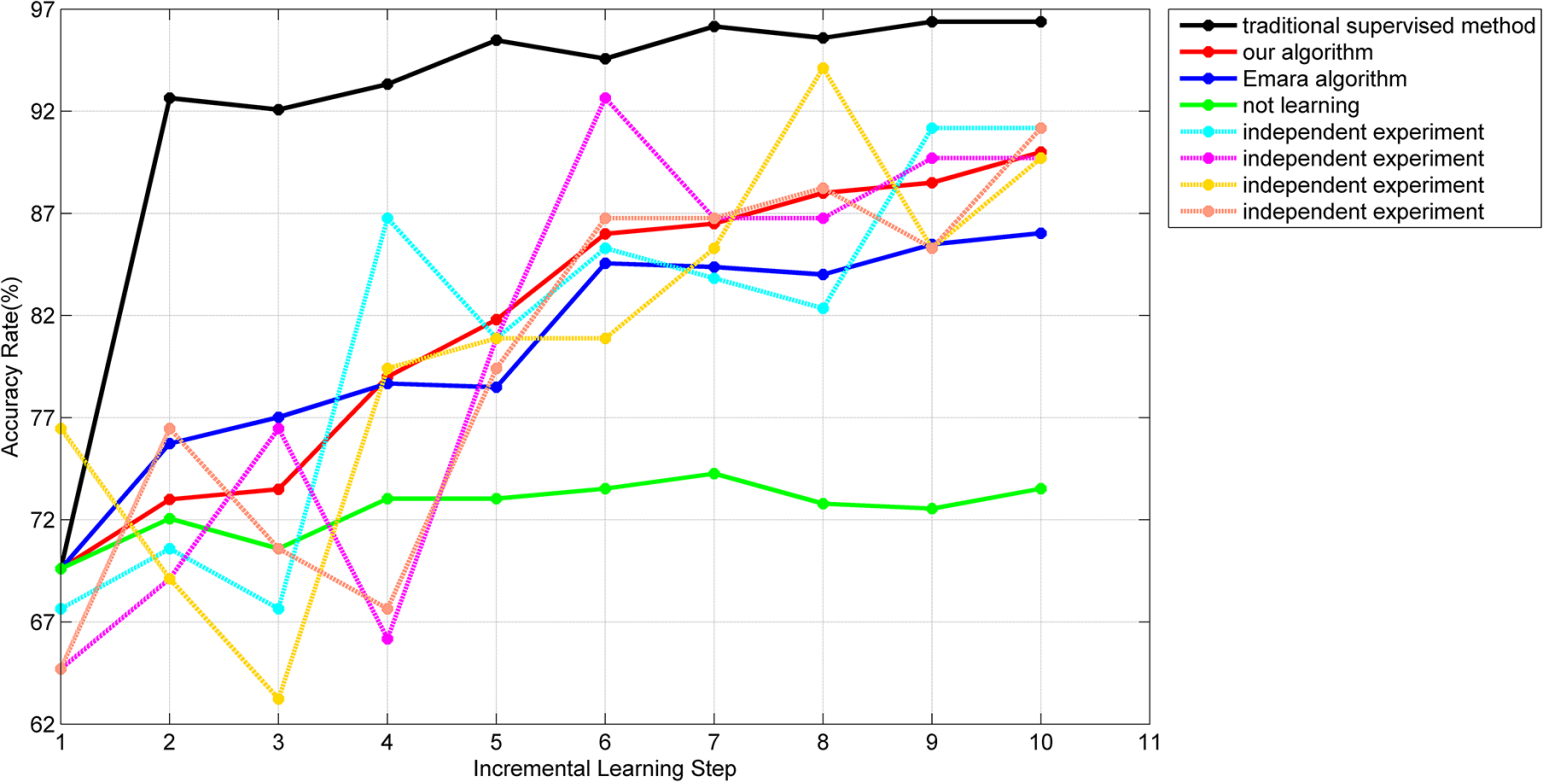
The accuracy rate of each new batch with different algorithms.

All the parameters and the normalization formula were the same as experiment 3.1. The experiment was also conducted many times and the accuracy rates of different algorithms were plotted in Fig 9.

In Fig 9, the processes of 4 independent experiments of our algorithm are represented with 4 dashed lines. The average process of 100 independent experiments of our algorithm, Emara’s algorithm [18] and the traditional supervised learning method are represented with the red, blue and black solid lines respectively. The average process with only the initial training set is represented with the green solid line.

As the non-face samples are changed into class B5 from class B1 and the strategy of our algorithm is relatively conservative in the “soft-start” phase, it can be seen from Fig 9 that the accuracy rate of our algorithm is low, which is similar to the green line and lower than the blue line, and that some large-scale fluctuations are presented in each independent experiment in the first 3 batches. However, with the main classifier obtaining more and more new semi-labeled samples and learning the new concept, our accuracy rate can rise to 80% and reach 90% finally, which is higher than the accuracy rate produced by the algorithm in [18], as Emara’s *S* ^3^ *VM* ^*new*^ is evaluated with a validation dataset which is not efficient in a changing environment and makes the algorithm more dependent on labeled samples to recover from past labeling mistakes, and proves that our algorithm can overcome “concept drift” successfully.

From the accuracy rate on the 8^th^, 9^th^ and 10^th^ batches of the red line, we can see that our data cleaning mechanism does not have a negative influence on the classifier but has a positive effect on reducing the amount of stored data. For the learning time, the average cost of our algorithm for the whole process is 12s while the average cost of Emara’s algorithm and the traditional method is 12s and 200s respectively, which shows the effectiveness of our algorithm. In addition, not any specific parameters and threshold values are set for the particular samples in the theoretical derivation, which makes the generalization performance of our algorithm excellent. The results of experiment 3.1 and experiment 3.2 also demonstrated at this point.

## Conclusions

This paper has proposed a novel classification algorithm based on the incremental semi-supervised SVM. Contributions of our algorithm are made as follows. First, our algorithm is applicable to the two different purposes of incremental learning. Second, our algorithm achieves similar performance in comparison to the traditional supervised learning approach but with smaller amount of stored data and learning time. Third, new unlabeled samples are effectively used in our algorithm to enrich the knowledge system of classifier with a combination of incremental learning and semi-supervised learning. Fourth, our algorithm is not dependent on the distribution assumption of problem structure, which makes it have an outstanding ability to overcome the concept drift in changing environments. Fifth, no specific parameters and threshold values are set in the theoretical derivation, which makes our algorithm display excellent generalization performance. Sixth, in order to extend the applications of our algorithm, the possible appearance of some new labeled samples is also considered during the learning process. All these advantages have been verified in the experiments. More research and practical applications about the incremental semi-supervised learning, such as incremental semi-supervised learning based on Adaboost, incremental semi-supervised online learning and incremental learning with possible mislabeled samples, would be carried out further in the future.

## Supporting Information

S1 Dataset MIT FACE. (ZIP)

S2 Dataset BMP2sn-c21_17_depression angle. (ZIP)

S3 Dataset BMP2sn-9563_17_depression angle. (ZIP)

S4 Dataset BMP2sn-9566_17_depression angle. (ZIP)

S5 Dataset T72sn-132_17_depression angle. (ZIP)

S6 Dataset T72sn-812_17_depression angle. (ZIP)

S7 Dataset T72sn-s7_17_depression angle. (ZIP)

S8 Dataset BMP2sn-c21_15_depression angle. (ZIP)
